# Barriers to the conduct and application of research in complementary and alternative medicine: a systematic review

**DOI:** 10.1186/s12906-017-1660-0

**Published:** 2017-03-23

**Authors:** Yasamin Veziari, Matthew J. Leach, Saravana Kumar

**Affiliations:** 1 School of Health Sciences, University of South Australia, North Terrace, Adelaide, SA 5000 Australia; 2 Department of Rural Health, University of South Australia, North Terrace, Adelaide, SA 5000 Australia

**Keywords:** Application, Barriers, Complementary and alternative medicine, Capacity, Culture, Conduct, Evidence-based practice, Research, Systematic review

## Abstract

**Background:**

The popularity of Complementary and alternative medicine (CAM) has grown considerably over the past few decades. This has been accompanied by increasing public pressure for CAM to be evidence-based. Notwithstanding, the conduct and application of research in CAM faces a number of obstacles. No systematic review has mapped these barriers to date. Therefore, this systematic literature review aimed to explore, identify and map the barriers to the conduct and application of research in CAM.

**Methods:**

Systematic searching of MEDLINE, Embase, AMED, CINAHL, The Cochrane library, Google scholar and Google was conducted between February and June 2016 for pertinent publications. Pearling (secondary searching) of retrieved publications was also undertaken. Literature published only in English were included; however, no year limit was placed for searching. Two critical appraisal tools were used to critically appraise descriptive studies and opinion publications.

**Results:**

A total of 21 eligible publications were included in this review; this comprised of eight primary research articles and thirteen opinion publications. A critical appraisal process found two categories of good quality publications while recognising their limitations in terms of descriptive and opinion publications. The synthesised data from the selected publications about the barriers to the conduct and application of research within CAM were captured within two broad components, namely capacity and culture*.* Capacity encompassed elements such as access, competency, bias, incentives and time. Encompassed within culture were elements relating to the values and complex system of CAM.

**Conclusions:**

Multiple barriers exist for the conduct and application of research in CAM. Given the growing popularity of these therapies, it is essential that the evidence base underpinning CAM also continues to expand. Without overt recognition of these barriers, enabling strategies cannot be applied. By addressing these barriers, CAM professions will be able to develop a critical mass and a well-coordinated research effort to assist the integration of evidence – based practice in CAM.

## Background

The use of Complementary and alternative medicine (CAM) has been steadily rising in Western countries where biomedical sciences have typically dominated the healthcare system [1, 2]. According to the World Health Organisation, there are an estimated 100 million users of CAM in Europe [3, 4]. In the United States of America (USA), close to 33.2 million US adults and children use some form of CAM [5]. High prevalence rates of CAM use are also reported in other developed countries, such as Australia [6], Korea [7], Canada [8], Singapore [9] and Japan [10].

Despite the growing popularity of CAM, there has been a renewed focus on the evidence-base of CAM [11, 12] with calls for CAM to demonstrate its effectiveness [13]. This focus for evidence in CAM has been increasingly talked about at a national and international level. For example, in the United States of America, The National Institutes of Health has established the National Center for Complementary and Integrative Health to carry out rigourous scientific investigation of CAM interventions [14]. In Australia, the National Health and Medical Research Council (NHMRC) conducted a series of reviews of CAM to determine their efficacy / effectiveness [15]. An important driver of this renewed focus on the effectiveness of CAM is Evidence Based Practice (EBP) [16].

EBP has been discussed in the medical literature for several decades [17], yet it is relatively new to CAM. Further, even though CAM has long valued empiricism as the foundation of CAM knowledge and skills [18–20], theoretical, philosophical and cultural differences have resulted in many CAM stakeholders being opposed to the EBP movement [21]. The reluctance to engage with EBP has resulted in ongoing scepticism towards CAM practices from stakeholders who represent mainstream health care [22–24]. Within CAM, while there has been a growing recognition for EBP [25], this has been constrained by a lack of reliable, trustworthy and diverse sources of research evidence [26, 27]. This can be attributed in part to CAM being a neglected area of research, with only small pockets of CAM research activity dispersed around the world [28].

Despite the growing popularity of CAM and the increasing need for evidence-based complementary and alternative medicine, the conduct of research (i.e. the systematic investigation of a phenomenon that serves to answer a specific research question) and application of research (i.e. the transference of research findings into clinical practice) in CAM continues to face a number of obstacles. However, no systematic review has mapped these barriers to date. Consequently, the aim of this systematic literature review was to explore, identify and map the barriers to conducting and applying research within CAM.

## Methods

### Study design

Systematic review of the literature and narrative synthesis.

### Aim

This systematic review set out to answer the following question: *What are the barriers to the conduct and application of research in Complementary and Alternative medicine?*

### Search strategy

The search strategy was guided by the Preferred Reporting Items for Systematic Reviews and Meta-Analyses guidelines [29]. The search was undertaken between February 2016 and June 2016. Prior to the commencement of a full search, a preliminary scoping search of CINAHL was undertaken to determine the feasibility of the review (i.e. extent of discussion on the topic) and to identify pertinent search terms. Once the search strategy was developed, it was independently checked and validated by an academic librarian at the University of South Australia.

The evidence gathering approach comprised two components: a comprehensive search of relevant databases and a search of references within eligible articles (i.e. pearling). The following databases were systematically searched (from their inception to June 2016) to identify relevant indexed publications: MEDLINE (Ovid), Embase (Ovid), AMED (EBSCO Host), CINAHL (EBSCO Host) and The Cochrane library. To avoid publication bias, a search of Google scholar and the Google search engine was undertaken to identify relevant grey literature. Pearling (secondary searching) was performed also, in which the bibliographies of included publications were screened for eligible articles. The search terms used for this review were as follows:

1. Complementary medicine OR Alternative medicine OR CAM
2. Research OR Evidence based practice
3. 1 AND 2

No restriction was placed on the year of publication; however, language (English) and human limits were applied.

### Selection criteria

The review included any publication(s) that explored barriers to the conduct or the application of CAM research; this was framed using the Population, Interest and Context (PICo) framework [30]. Table 1 provides an overview of the PICo components and the review selection criteria.

**Table 1:** Overview of review selection criteria

Construct	Justification
Population	Inclusion criteria:
	- CAM researchers with or without CAM background
	- CAM practitioners/clinicians
	Exclusion criteria:
	- CAM users/non users
	- CAM products
Interest	Inclusion criteria:
	- Research that looks at the barriers to the conduct or application of research in CAM
	- Opinion publications with a reference list
	- Studies or publications on EBP in CAM
	Exclusion criteria:
	- Primary or secondary research that investigates the effectiveness or efficacy of CAM
	- Economic evaluations of CAM
	- Opinion articles without adequate references
	- Promotional materials
	- Methodological studies
Context	Inclusion criteria:
	- Research (conduct or application of)
	Exclusion criteria:
	- Publications reporting the prevalence of CAM use
	- Publications describing CAM

*CAM* Complementary and alternative medicine, *EBP* Evidence-based practice

#### Population

With regards to the conduct of research, publications had to focus primarily on CAM researchers; this could include researchers from other health disciplines (such as medicine) who were undertaking CAM research. In terms of the application of research, the core focus of the publication had to be CAM practitioners, from any CAM discipline. Any publications focusing on consumer perspectives were excluded.

#### Interest

The key interest of this review was the barriers to the conduct or application of research in CAM. The focus of this research was not about effectiveness, thus any research testing the effectiveness or efficacy of an intervention were excluded. Also excluded were publications discussing research methodology or cost effectiveness.

#### Context

The context of the review was complementary and alternative medicine. Any research that focused on describing CAM or CAM use was excluded.

### Publication type

The review included any primary research article (quantitative and qualitative) or opinion publication (commentary, narrative and expert opinion). Secondary research (reviews of the literature), newsletters or opinion publications without references or a publication date were excluded.

### Selection of included publications

The title and abstract of each listed publication was screened by YV. Potentially eligible publications were then retrieved as full text to determine if the publication addressed the review question and met the review inclusion criteria. A preliminary list of potentially relevant publications was subsequently generated and reviewed by each author. The authors discussed each publication until consensus was reached on the final list of included articles.

### Critical appraisal

Each included paper was appraised by at least two authors, with the quality of publications assessed using one of two critical appraisal tools. The Narrative, Opinion and Text Assessment and Review Instrument (NOTARI) [30] was used to appraise opinion publications. This instrument contains seven criteria, which enable judgement of the source of the opinion, expertise of the authors, main focus of the article, logic of the argument, analytical development of the argument, references to literature, and peer support of opinion. Items receiving a ‘yes’ response were assigned one mark, while a ‘no’ response received zero marks. Total scores for NOTARI range from 0 to 7.

The Critical Appraisal of a Survey toolkit [31] was used to appraise primary research articles reporting on survey research. This toolkit contains twelve criteria, which assess the clarity of the question/issue, appropriateness of the study design, method of subject selection, possibility of sampling bias, representativeness of the study sample, pre-study considerations of sample size, response rate, validity and reliability of the questionnaire, reporting of statistical significance, reporting of confidence intervals, consideration of confounding factors, and the applicability of the results. Each criterion was assessed as either yes (criterion met), can’t tell or no (criterion not met), with a score of 1, 0 and 0 assigned, respectively. Total scores for the toolkit range from 0 to 12, with a score of 9 or more considered excellent qualities [32].

### Data extraction

A customised data extraction tool was developed to extract information relevant to the aim of this review. Data extracted included the author name, year of publication, publication type / study design, country of study, survey tool used (if appropriate), domains of measurement (if appropriate), barriers to the conduct of research, and barriers to the application of research.

### Data analysis

Due to the descriptive nature of this review, the analysis of the included studies was undertaken in a narrative manner. Following data extraction, barriers to the conduct and application of research in CAM were reviewed independently by each author to identify subthemes (i.e. low-level themes). The authors then convened to collaboratively refine the subthemes, and to identify overarching themes common across all studies (i.e. high-level themes). The team deliberated until consensus was reached on the themes and sub-themes for the barriers to both the conduct and application of research in CAM.

## Results

**Fig. 1:**
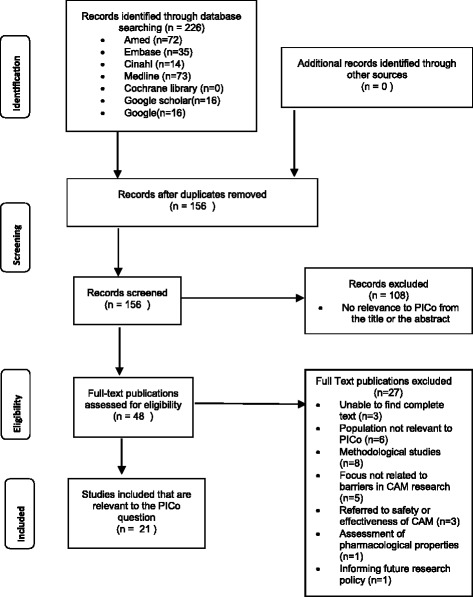
PRISMA 2009 Flow Diagram

Figure 1 provides an overview of the literature selection process. The search identified a total of 226 publications. Following the removal of 70 duplicate publications and 108 irrelevant papers, 48 publications remained. Of these, 27 papers were excluded as they did not meet the review selection criteria. This resulted in a total of 21 studies being included in this review.

### Overview of included studies

The search identified two categories of publications. The first category comprised eight primary research publications (e.g. cross-sectional studies). The second category encompassed thirteen opinion publications.

The eight primary research articles originated from three countries, including Australia (*n* = 3), United States of America (USA) (*n* = 4) and Canada (*n* = 1). The majority of studies were conducted in the past three years, with three studies conducted in 2015 and three in 2013; the remaining two studies were conducted between 2009-2011. With regards to participants, five studies focused specifically on chiropractors, two studies focused on CAM practitioners more generally, and one involved a combination of CAM and conventional health practitioners. The evidence-based practice attitude and utilization survey (EBASE tool; [33]) was commonly used across these studies (*n* = 5), with four studies modifying the tool to suit individual research requirements. Of the eight primary research studies, seven studies used a cross-sectional study design, and one was a descriptive analysis of National Health and Medical Research Council grant data [34]. Table 2 provides an overview of the characteristics of the primary research articles included in this review.

**Table 2:** Characteristics of included research studies^a^

Author/ Year	Design	Country	Participants/Sample size/Response rate	Survey Tool used	Domains of measurement
Alcantara & Leach 2015 [36]	Cross-sectional study	USA	Chiropractors.	EBASE (modified)	EBP related skills
			*n* = 500		Attitudes towards EBP
			Response rate = 32.4%		
					Barriers
Bussières et al. 2015 [37]	Cross-sectional study	Canada	Chiropractors.	EBASE (modified)	Attitudes towards EBP
			*n* = 7200		Knowledge/Skills in EBP
			Response rate = 7.7%		
					EBP training/Education
					Use of EBP
					Barriers to EBP
					Facilitators of EBP uptake
Leach & Gillham 2011 [50]	Cross-sectional study	Australia	CAM practitioners.	EBASE	Attitude towards EBP
			*n* = 400		Knowledge/Skills in EBP
			Response rate = 36%		
					EBP training/Education
					Use of EBP
					Sources of Information used to inform clinical decisions
					Barriers to EBP
					Facilitators of EBP uptake
Roecker et al. 2013 [53]	Cross-sectional study	USA	Chiropractors.	EBASE (modified)	Skill level in EBP
			*n* = 309		Extent of EBP in clinical practice within the last month
			Response rate = 48%		
					Sources of information to inform clinical practice
					Barriers to EBP in clinical practice
Schneider et al. 2015 [51]	Cross-sectional study	USA	Chiropractors.	EBASE (modified)	EBP Training /Education
			*n* = 30,000		Attitudes towards EBP
			Response rate = 4.4%		
					Skills in EBP
					Use of EBP
					Barriers to EBP
					Facilitators of EBP uptake
Tilburt et al. 2009 [52]	Cross-sectional study	USA	Acupuncturists, naturopaths, internists and rheumatologists.	Self-administered questionnaire	Awareness of CAM trials
					Skills to interpret research results
					Attitude towards research results
			*n* = 2400		
			Response rate = 65%		
Walker et al. 2013 [38]	Cross-sectional study	Australia	Chiropractors.	Jett et al. questionnaire (modified)	Attitudes towards EBP
			*n* = 4378		Motivation to engage in EBP
			Response rate = 13%		
					EBP training/Education
					Using literature in clinical decision-making
					Availability and ability to access information
					Barriers to EBP
Wardle & Adams 2013 [34]	Descriptive research	Australia	CAM practitioners.	N/A	Review of grants awarded by NHMRC towards CAM research

*EBASE* Evidence-based practice attitude and utilization survey

^a^Excludes opinion publications as there is limited information to report

In relation to the thirteen opinion publications, the countries of origin were USA (*n* = 6), Australia (*n* = 4) and UK (*n* = 4). Unlike the primary research studies, opinion publications originated as early as 1999, with the most recent published in 2015. Most of the opinion publications were published in early 2000, which indicates more than a decade of interest in this topic.

### Critical appraisal of included studies

The results of the critical appraisal are presented in two distinct sections. The first section focuses on the conduct of research. The second section focuses on the application of research findings.

#### Critical appraisal of studies reporting barriers to the conduct of research in CAM

Table 3 provides an overview of the critical appraisal scores of the fourteen publications reporting on the barriers to the conduct of research in CAM. The overall methodological quality of all included articles was good, scoring 6/7, with one article having achieved the lowest score of 5/7 [35]. The one criterion that was consistently not met was criterion three, which ascertained if the focus of the article was the patient. Given that the focus of the research was CAM practitioners and researchers, and not patients, all studies did not score for this particular criterion. The Rosner 35 study lost an additional mark because it did not meet the following criterion: ‘argument was developed analytically’ - primarily, because it was a testimonial stating facts about the barriers and obstacles to CAM research.

**Table 3:** Critical appraisal scores for publications reporting on the barriers to the conduct of research in CAM

Study	Database	Criterion 1	Criterion 2	Criterion 3	Criterion 4	Criterion 5	Criterion 6	Criterion 7	Total Score
Ahn et al. 2010 [45]	Embase	Y	Y	N/A	Y	Y	Y	Y	6/7
Bensoussan & Lewith 2004 [39]	Embase	Y	Y	N/A	Y	Y	Y	Y	6/7
Ernst 1999 [40]	Amed	Y	Y	N/A	Y	Y	Y	Y	6/7
Ernst 2003 [43]	Google scholar	Y	Y	N/A	Y	Y	Y	Y	6/7
Evans 2007 [47]	Google	Y	Y	N/A	Y	Y	Y	Y	6/7
Giordano, Engebretson & Garcia 2005 [44]	Embase	Y	Y	N/A	Y	Y	Y	Y	6/7
Jonas 2005 [42]	Amed	Y	Y	N/A	Y	Y	Y	Y	6/7
Lewith & Holgate 2000 [41]	Amed	Y	Y	N/A	Y	Y	Y	Y	6/7
Long & Mercer 1999 [49]	Amed	Y	Y	N/A	Y	Y	Y	Y	6/7
Nahin & Strauss 2001 [48]	Google scholar	Y	Y	N/A	Y	Y	Y	Y	6/7
Rosner 2000 [35]	Cinahl	Y	Y	N/A	Y	N	Y	Y	5/7
Shekelle et al. 2005 [46]	Amed	Y	Y	N/A	Y	Y	Y	Y	6/7
Steel & McEwen 2014 [25]	Cinahl	Y	Y	N/A	Y	Y	Y	Y	6/7
Wardle & Adams 2013 [34]	Embase	Y	Y	N/A	Y	Y	Y	Y	6/7

Legend:

Criterion 1. Is the source of the opinion clearly identified?

Criterion 2. Does the source of the opinion have standing in the field of expertise?

Criterion 3. Are the interests of patients/clients the central focus of the opinion?

Criterion 4. Is the opinions basis in logic/experience clearly argued?

Criterion 5. Is the argument developed analytical?

Criterion 6. Is there reference to the extant literature/evidence and any in congruency with it logically defended?

Criterion 7. Is the opinion supported by peers?

Yes = Y = 1; No = N = 0; Unclear = UC = 0

#### Critical appraisal of studies reporting barriers to the application of research findings

Table 4 provides an overview of the critical appraisal scores for the seven primary research studies that had a specific focus on the application of research findings in CAM. Overall, the methodological quality of the surveys was good as the lowest score was 9/12 (the highest being 12/12). Generally, three criteria were commonly not addressed across the studies; these related to sampling and selection bias, power calculation and response rate. Sampling and selection bias was often evident when the survey instrument was distributed electronically through email only; this meant those who did not have ready access to internet or email were automatically excluded. This was the case for Alcantara and Leach [36] and Bussières et al. [37]. Some surveys did not provide evidence of power calculations prior to the start of their research (such as Alcantara & Leach) [36] or had poor response rates, some as low as 13% (Walker et al.) [38].

**Table 4:** Critical appraisal scores for studies reporting on the barriers to the application of research findings in CAM

Study	Database	Criterion 1	Criterion 2	Criterion 3	Criterion 4	Criterion 5	Criterion 6	Criterion 7	Criterion 8	Criterion 9	Criterion 10	Criterion 11	Criterion 12	Total Score
Alcantara & Leach 2015 [36]	MEDLINE	Y	Y	Y	CT	Y	CT	N	Y	Y	Y	Y	Y	9 / 12
Bussières et al. 2015 [37]	MEDLINE	Y	Y	Y	N	Y	Y	Y	Y	Y	Y	Y	Y	11 / 12
Leach & Gillman 2011 [50]	CINAHL	Y	Y	Y	Y	Y	Y	Y	Y	Y	Y	Y	Y	12 / 12
Roecker et al. 2013 [53]	MEDLINE	Y	Y	Y	Y	Y	CT	Y	Y	Y	Y	Y	Y	11 / 12
Schneider et al. 2015 [51]	MEDLINE	Y	Y	Y	CT	Y	CT	Y	Y	Y	Y	Y	Y	10 / 12
Tilburt et al. 2009 [52]	Embase	Y	Y	Y	Y	Y	Y	Y	y	Y	Y	Y	Y	12 / 12
Walker et al. 2013 [38]	MEDLINE	Y	Y	Y	Y	Y	Y	N	Y	Y	Y	Y	Y	11 / 12

Legend:

Criterion 1. Did the study address a clearly focused question/issue?

Criterion 2. Is the research method (study design) appropriate for answering the research question?

Criterion 3. Is the method of selection of the subjects (employees, teams, divisions, organisations) clearly described?

Criterion 4. Did the sampling strategy avoid selection bias?

Criterion 5. Was the sample of subjects representative with regard to the population to which the findings referred?

Criterion 6. Was the sample size based on pre-study considerations of statistical power?

Criterion 7. Was a satisfactory response rate (≻40%) achieved?

Criterion 8. Are the measurements (questionnaires) likely to be valid and reliable?

Criterion 9. Were appropriate statistical tests applied?

Criterion 10. Are confidence intervals given for the main results?

Criterion 11. Have the confounding factors been accounted for?

Criterion 12. Can the results be applied to your organisation?

Yes = Y; Cannot tell = CT; No = N

### Overview of barriers

The synthesised findings are presented in two parts – barriers that influenced the conduct of research in CAM, and barriers that influenced the application of research findings in CAM. While there were barriers unique to each group, there were also barriers common to both groups.

#### Barriers to the conduct of research findings

Table 5 provides an overview of the fourteen publications that discussed the barriers to the conduct of research in CAM. Broadly, these barriers were captured within one of two categories: *capacity and culture.* The category *“capacity”* encompassed barriers that influenced the conduct of research and could be amenable to change with concerted effort and resources. The category *“culture”* contained barriers that influenced the conduct of research and were not readily amenable to change.

**Table 5:** Barriers to the conduct of research

First author, year	Publication type OR study design	Country	Discipline focus OR General CAM	Capacity			Culture	
				Access	Competency	Bias	Values	Complex system
Ahn et al. 2010	Opinion	USA	General CAM	●	●			●
Bensoussan & Lewith 2004	Opinion	Australia	General CAM	●	●	●	●	●
Ernst 1999	Opinion	UK	General CAM	●	●	●	●	
Ernst 2003	Opinion	UK	General CAM	●		●	●	●
Evans 2007	Opinion	Australia	General CAM	●	●	●	●	●
Giordano, Engebretson & Garcia 2005	Opinion	USA	General CAM	●	●	●	●	●
Jonas 2005	Opinion	USA	General CAM	●	●	●	●	●
Lewith & Holgate 2000	Opinion	USA	General CAM	●	●	●	●	●
Long & Mercer 1999	Opinion	UK	General CAM	●		●	●	●
Nahin &Strauss 2001	Opinion	UK	General CAM	●		●	●	●
Rosner 2000	Opinion	USA	General CAM	●	●	●		
Shekelle et al. 2005	Opinion	USA	General CAM	●		●		●
Steel & McEwen 2013	Opinion	Australia	General CAM	●	●	●	●	
Wardle & Adams 2013	Descriptive Research	Australia	General CAM	●		●	●	●

Within the *“capacity”* category, three sub-categories of barriers were identified; this included “*access”, “competency” and “bias”.* “Access” related to barriers such as lack of access to funding, training / skills in research, CAM journals in mainstream databases, quality research and quality researchers in CAM. All the included studies reported on access barriers. Bensoussan and Lewith [39] stated that since 2001, only 0.085% ($850,000) of about $1 billion of National Health and Medical Research Council research funding was allocated to CAM research in Australia. Ernst [40] added to this, highlighting that research funding schemes are not designed to support CAM research and the dearth of university researchers interested in CAM does not warrant funding/support [41]. Poor access to funding contributed to a lack of research training [42], lack of accessibility of well-trained scientists entering CAM [43], few opportunities for research education and research in CAM [44] and finally, a paucity of grant applications [25, 39, 41]. Access barriers also extended to the lack of access to well-qualified CAM researchers due to the lack of incentives [41, 43]. Other barriers included poor access to high quality systematic reviews [41], limited qualitative studies [25] and lack of access to CAM-centric diagnostic research [45]. Shekelle et al. [46] also highlighted that the inconsistent keywords, descriptors, subjects and differing indexing procedures across databases pose a challenge in locating CAM research.

Nine articles reported on barriers related to the sub-category “competency”; this referred to the skills, knowledge and competency of the CAM practitioner in the conduct of research. There were a range of different issues that were captured within the included articles, such as insufficient training/literacy in research [25, 42], inadequate research experience, limited ability to interpret results [42, 44], ignorance regarding research methodologies [41] and lack of awareness of research being undertaken [40]. Some authors indicated that CAM practitioners did not have a strong research background [44] and thus, were not well qualified to conduct research [35, 39]. Lewith and Holgate [41] highlighted that few researchers who were investigating CAM topics had explicit CAM experience or knowledge and conversely most CAM therapists did not have a strong background in research.

The sub-category *“bias”* was an important barrier reported in thirteen publications; this related to the inherent negative perceptions about CAM research. This was well captured by Jonas [42] who stated, *“Another major challenge to CAM research comes from the underlying assumptions of many CAM practices.”* These negative perceptions were identified across a range of areas including the medical community, medical institutions, universities, funding agencies and mainstream databases. The mainstream medical community was reported to discredit CAM and CAM research [35], with collaboration between CAM and mainstream medical scientists/practitioners lacking [44]. This was argued to have resulted in poor engagement between mainstream and CAM researchers with no opportunities for CAM professions to develop a research profile [25, 34]. The publications also pointed to a perceived bias in terms of access to research funding for CAM; this was attributed to non-recognition of CAM as a research priority [47], limited understanding of CAM research and hence negative attitudes of grant reviewers [34, 39, 48] and insufficient interest in CAM by researchers that deterred CAM research funding [41]. Some authors also highlighted bias in CAM publications due to the lack of specialist CAM reviewers, including a lack of understanding of CAM modalities [35, 46].

Within the *“culture”* category, two sub-categories of barriers were identified; these were *“values”* and *“complex systems”.* The sub-category *“values”* related to a range of historical and philosophical perspectives, which underpinned CAM as a unique and stand-alone discipline. Some authors reflected that CAM did not fit within the mainstream biomedical model of care [42] and that CAM practitioners have a different philosophical approach than traditional medicine. This was exemplified by Giordano, Engebretson and Garcia [44], who stated that complementary medicines “do not fit easily into the mainstream biomedical conceptualization of mechanism, scope/nature of treatment, or the role of the clinician – patient interaction”. This perceived uniqueness of CAM was seen to contribute to a reluctance of CAM to engage with mainstream research [34, 39–41] or to exchange research information [42].

Another common cultural barrier was the CAM educational model. Much of CAM education was reported as being undertaken in private colleges, and not in university settings, with limited postgraduate research-focused opportunities [25, 44]. As such, CAM education had a predominant focus on clinical practice rather than research. CAM practitioners who were interested in research had to navigate outside their undergraduate training [25], which posed significant barriers.

The sub-category “*complex system*” related to the complexity underpinning CAM research, which may not be readily captured in the mainstream research framework. The “*complex systems*” barriers were reported in eleven articles and were inclusive of the patient-practitioner relationship, the care and treatment model, complex interventions and challenges in research design and execution. Authors highlighted that CAM involves significant and extended patient-practitioner interaction [39, 45]; as such, it was difficult to deconstruct the patient-practitioner relationship [45]. A number of articles reported on CAM having a holistic treatment approach [39] where patients were treated as individuals, and consequently, a combination of treatments were used both within and between patients [42, 48]. In other words, the CAM health and healing approach was seen to be different from the biomedical model [44, 49], and was generally not well-understood [44]. Given these complexities, several allied barriers to conducting research were identified, including an inability to undertake blinding and provide a true control/placebo group [44, 45], limited generalisability of findings, and associated funding [43]. These issues were best captured by Long and Mercer [49], who stated that “not only are there important differences between individual CAMs, but there are sometimes further significant divisions within a CAM”.

#### Barriers to the application of research findings

Table 6 provides an overview of the eight articles (including seven primary research studies and one opinion publication) that identified the barriers to the application of research findings by CAM practitioners within their practices. These barriers could be collapsed into two broad categories: *capacity* and *culture*. Similar to the barriers to conducting research, the category “capacity” encompassed barriers that influenced the application of research findings and could be amenable to change with concerted effort and resources. The category “culture” contained barriers that influenced the application of research but were not readily amenable to change.

**Table 6:** Barriers to the application of research

First Author, year	Publication type OR Study Design	Country	Discipline focus OR General CAM	Capacity					Culture
				Access	Competency	Bias	Incentive	Time	
Alcantara & Leach 2015 [36]	Cross-sectional study	USA	Chiropractic	●	●			●	●
Bussières et al. 2015 [37]	Cross-sectional study	Canada	Chiropractic	●	●		●	●	●
Evans 2007 [47]	Opinion publication	Australia	General CAM	●	●				
Leach & Gillham 2011 [50]	Cross-sectional study	Australia	General CAM	●	●				●
Roecker et al. 2013 [53]	Cross-sectional study	USA	Chiropractic	●				●	
Schneider et al. 2015 [51]	Cross-sectional study	USA	Chiropractic	●	●		●	●	●
Tilburt et al. 2009 [52]	Cross-sectional study	USA	General CAM		●				●
Walker et al. 2013 [38]	Cross-sectional study	Australia	Chiropractic	●	●	●			●

Within the category *“capacity”* were five sub-categories. The sub-category *“access”* related to the accessibility of research. This included access to research training and research skills [36, 37, 50, 51], and lack of training on how to implement research findings into practice [52]. Barriers to access also extended to other concepts such as limited access to resources [38] and CAM researchers [37], lack of access to research that supported EBP [36, 41, 53] and lack of access to high quality research evidence that can be readily translated to practice [36, 37, 47, 50, 53].

The subcategory *“competency”* focused on the competency, skills and knowledge of the CAM practitioner in relation to research. Seven studies identified issues such as the lack of knowledge and skills in locating evidence [52], critical appraisal of evidence [37], interpreting results [37], lack of awareness of practice guidelines and their availability [38] and presenting evidence to patients [47].

The sub-category *“bias”* focused on the (often negative) perceptions of research within CAM. For example, Walker et al. [38] identified that older chiropractors were less likely to agree to the application of research findings into practice. This may be because their past training might not have had a specific focus on EBP and may have had a considerable focus on the historical/traditional values of CAM. This may have resulted in these chiropractors forming an antithesis viewpoint of EBP.

The two research studies that explored chiropractors’ perspectives of the application of research findings into practice - both of which were undertaken in North American settings – identified the lack of *“incentive”* (another sub-category) as a critical barrier. Chiropractors in the Bussières et al. [37] and Schneider et al. [51] studies reported that there was no financial or tangible incentive for them to undertake EBP in their local settings. Similarly, *“time”,* another sub-category, was a commonly reported barrier to the application of research findings into practice. Four studies [36, 37, 51, 53] highlighted that due to limited time, chiropractors may focus on clinical priorities during the consultation rather than accessing and applying evidence into practice.

The category *“culture”* captured a number of barriers related to the general beliefs, attitudes and behaviours of practitioners within the CAM professions. Research by Alcantara et al. [36], Bussières et al. [37], Leach and Gillham [50], Schneider et al. [51], Tilburt et al. [52] and Walker et al. [38] cite entrenched cultural barriers in CAM whereby there was a distinct lack of interest in research, irregular access to the research literature, and infrequent use of databases to inform clinical practice. These barriers could be regarded as profession-wide issues due to the lack of professional and peer support for research, and the over-reliance on anecdotes, expert opinion and traditional evidence. Patient perspectives also contributed to this category in the form of discrepancy between patient expectations and research evidence.

## Discussion

The purpose of this systematic review was to identify the barriers to the conduct and application of research in CAM. While evidence-based practice has been increasingly integrated within the mainstream health professions, within the CAM professions, use of EBP to inform clinical decision making still remains in its infancy. This is particularly concerning as there is widespread and growing use of CAM within the community [39]. Recently, the importance of evidence-based CAM has been highlighted in Australia, with the NHMRC undertaking a review of evidence across a number of CAM disciplines, including homeopathy [15]. Even within CAM, many researchers have called for a greater emphasis on EBP within the CAM professions [42, 50, 53].

**Fig. 2:**
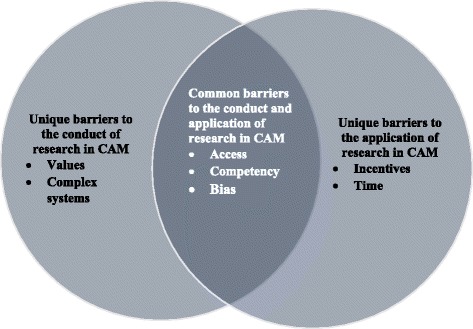
Overview of the common and unique barriers to the conduct and the application of research in CAM

While the use of EBP in CAM could be considered as a given, an important problem confronts CAM stakeholders wanting to engage with EBP. An important component of EBP is research evidence and this systematic review has identified numerous barriers to the conduct and application of research evidence in CAM. Findings from this review suggest that there are common and unique barriers to the conduct and application of research in CAM (Fig. 2). Barriers common to both areas were “access” “competency” and bias”.

While this was the first systematic review to map the barriers to the conduct and application of research in CAM, similar findings have been reported in other areas of health [54–56]. In particular, there are a number of research studies in health disciplines such as physiotherapy [57], occupational therapy [58], podiatry [59], speech and language therapy [60], social work [61] and nursing [62, 63]. These findings suggest that, irrespective of the discipline, there are some shared common barriers. However, it is poignant to note that one cardinal difference between CAM professions and other health disciplines, is that many of the barriers for other health disciplines have a predominant focus on the application of research findings into practice whereas for CAM, these barriers seem to extend to the conduct as well as the application. One possible explanation for this is that for many health disciplines, over time, the research evidence has been well established. However, for the field of CAM, the development of research evidence is still in its infancy and continues to evolve. Due to this, these barriers extend to the conduct as well as the application of research in CAM.

There are a number of additional factors that may impact the conduct and application of research in CAM, which were not identified in this review. For example, there is disagreement within the CAM research community on what constitutes best research evidence for CAM stakeholders [64]. Historical biases such as the negative perception of CAM, access to funding options, and traditional values held close to CAM practitioner models of care have also been reported in the literature [27]. Poorly reported studies create a lack of accurate and accessible information, which suggests that CAM researchers (and CAM journals) may not be aware of, or adhere to, international research reporting standards [27]. Additionally, there exist multiple factors that create a divide between CAM research and application, the most prominent being the ability of CAM practitioners to translate research evidence into practice [65]. Claims of a culture of ‘anti-science’ anti-medicine’ and ‘anti-establishment’ in CAM have also been suggested as contributing to the research-practice gap in the field [65].

While there is no one magic bullet to overcome these myriad of factors, a range of strategies could be implemented to facilitate the conduct and application of research within the CAM community. Such tactics might include increasing knowledge through investigator-driven funding, lobbying for unbiased grant review processes, integrating research training into undergraduate CAM programs, and establishing methods for identifying and assessing evidence with ongoing efforts to dispel the myths about CAM. These strategies have been trialled with other professions, such as physiotherapy, with success. Over the course of the past few decades, physiotherapy has moved on to embrace the importance of research to inform its clinical practice [66]. While acknowledging the role of research evidence, physiotherapy continues to recognise the importance of getting the balance right between research evidence, clinical practice and patient morals, values and beliefs [67].

One of the strategies adopted by physiotherapy has been to embed research within the undergraduate curricula, thereby creating a critical mass of physiotherapists who are familiar with, and have skills to, embed research evidence in clinical practice. Further, there has been the establishment of a dedicated research grant funding body [68] for which physiotherapists can apply for funding to conduct physiotherapy-related research [68]. Physiotherapists have also successfully tapped in mainstream health and medical research grants, which have historically been accessed by medical disciplines. For example, since 2000, there has been a steady increase in the number of National Health and Medical Research Council funded grants with one or more physiotherapists as chief investigators [69]. These are just a few strategies that have addressed the barriers of access, competency and bias in physiotherapy, which the CAM professions can perhaps learn from if it endeavours to overcome the barriers to conducting and applying research in CAM.

As with any research, this systematic review too has limitations. These limitations include language bias (as only English-language studies and articles were included), use of opinion publications (due to the nature of the review question and lack of research studies) and the inclusion of studies with methodological bias (i.e. the possible presence of sampling and selection bias; inadequate reporting of power calculation; low response rates). Despite these limitations, a comprehensive search strategy, underpinned by a systematic approach, have ensured access to the current best available evidence to answer the review question.

## Conclusion

### Implications for practice

While it is clear that CAM professions must engage with research, operationalising this in practice continues to face barriers. Without explicit recognition of these barriers, enabling strategies cannot be implemented. These enabling strategies may include dedicated access to research funding for CAM, fellowship opportunities for emerging CAM researchers and clinicians, embedding research as part of undergraduate training in CAM and improved access to ongoing continuous professional development opportunities for clinicians in the areas of EBP and research. By doing so, CAM professions can build a critical mass and a well-coordinated research effort that will assist in integrating EBP in CAM.

### Implications for research

Despite the availability of research on the barriers to the conduct and application of research in CAM, much of this research effort is ad hoc and opportunistic. To date there are no structured means of capturing and measuring the barriers to the conduct and application of research in CAM. Future research should focus on systematically mapping these barriers, through the use of psychometrically rigorous instruments and methodologically sound research. Mapping of these barriers could assist in promoting the importance of engaging with research in CAM, develop and trial enabling strategies and identify what strategies works for whom in which contexts.

## Abbreviations

CAM: Complementary and alternative medicine
EBP: Evidence-based practice
NOTARI: The narrative, opinion and text assessment and review instrument
PICo: Population, interest and context
